# Inhibition of cysteine-serine-rich nuclear protein 1 ameliorates ischemia–reperfusion injury during liver transplantation in an MAPK-dependent manner

**DOI:** 10.1186/s43556-024-00185-z

**Published:** 2024-06-21

**Authors:** Zhoucheng Wang, Wenwen Ge, Xinyang Zhong, Shizheng Tong, Shusen Zheng, Xiao Xu, Kai Wang

**Affiliations:** 1 NHC Key Laboratory of Combined Multi-organ Transplantation, Hangzhou, 310003 Zhejiang China; 2 Zhejiang University School of Medicine, Hangzhou, 310058 Zhejiang China; 3 The Fourth School of Clinical Medicine, Zhejiang Chinese Medical University, Hangzhou, 310053 Zhejiang China; 4 Department of Hepatobiliary and Pancreatic Surgery, Shulan Hospital of Hangzhou, Hangzhou, 310022 Zhejiang China; 5 Department of Hepatobiliary & Pancreatic Surgery and Minimally Invasive Surgery, Zhejiang Provincial People’s Hospital (Affiliated People’s Hospital), Hangzhou Medical College, Hangzhou, 310024 Zhejiang China; 6 Institute of Translational Medicine, Zhejiang University, Hangzhou, 310000 Zhejiang China

**Keywords:** Liver transplantation, Hepatic ischemia–reperfusion injury (HIRI), Cysteine-serine-rich nuclear protein-1 (CSRNP1), Mitogen-activated protein kinases, Nanoparticles

## Abstract

Hepatic ischemia–reperfusion injury (HIRI) is a critical pathophysiological process during liver transplantation (LT). Multiple genes and signal pathways are dysregulated during HIRI. This study aims to identify genes as potential therapeutic targets for ameliorating HIRI. Datasets containing samples from the human donor liver (GSE151648) and mouse HIRI model (GSE117066) were analyzed to determine differentially expressed genes (DEGs). The selected DEGs were confirmed by real-time PCR and western blot in the hepatocyte hypoxia-reoxygenation (HR) model, mouse HIRI model, and human liver samples after transplantation. Genetic inhibition was used to further clarify the underlying mechanism of the gene in vitro and in vivo. Among the DEGs, *CSRNP1* was significantly upregulated (|log FC|= 2.08, *P* < 0.001), and was positively correlated with the MAPK signal pathway (*R* = 0.67, *P* < 0.001). CSRNP1 inhibition by siRNA significantly suppressed apoptosis in the AML-12 cell line after HR (mean Annexin^+^ ratio = 60.62% vs 42.47%, *P* = 0.0019), but the protective effect was eliminated with an additional MAPK activator. Knocking down *CSRNP1* gene expression by intravenous injection of AAV-shRNA markedly reduced liver injury in mouse HIRI model (ALT: AAV-NC vs AAV-shCsrnp1 = 26,673.5 ± 2761.2 vs 3839.7 ± 1432.8, *P* < 0.001; AST: AAV-NC vs AAV-shCsrnp1 = 8640.5 ± 1450.3 vs 1786.8 ± 518.3, *P* < 0.001). Liver-targeted delivery of siRNA by nanoparticles effectively inhibited intra-hepatic genetic expression of *Csrnp1* and alleviated IRI by reducing tissue inflammation and hepatocyte apoptosis. Furthermore, CSRNP1 inhibition was associated with reduced activation of the MAPK pathway both in vitro and in vivo. In conclusion, our results demonstrated that CSRNP1 could be a potential therapeutic target to ameliorate HIRI in an MAPK-dependent manner.

## Introduction

Hepatic ischemia–reperfusion injury (HIRI) is an inevitable complication during the process of liver transplantation (LT) [1]. Oxidative stress initiates ischemic injury and leads to a sterile inflammation following blood reperfusion that aggravates the liver injury [2]. Moreover, activation of innate immunity could further aggravate liver injury [3]. These combined effects of stress and inflammation ultimately caused hepatocyte apoptosis and liver dysfunction [4]. HIRI may result in early allograft dysfunction (EAD), primary nonfunction (PNF), and even graft failure during the acute phase after LT, which may then affect prognosis in patients [5]. Although many efforts have been made to protect the liver from injury, there is still no effective drug to prevent HIRI, and the incidence of EAD reached up to 30% [6]. Revealing the underlying mechanisms and facilitating mitigation strategies are of great importance for improving post-transplant outcomes [7].

The mitogen-activated protein kinases (MAPKs) are a group of highly conserved serine/threonine protein kinases, which exert a pivotal role in development, tumorigenesis, and damage repair [8, 9]. Activated MAPKs, including phosphorylated c-Jun N-terminal kinase (JNK/SAPK), extracellular signal-regulated kinase (ERK/P44/42), and P38, enter the nucleus to regulate transcription of the downstream genes associated with cell survival, proliferation and apoptosis [10]. Importantly, MAPKs exert a critical effect on extra-hepatic organ ischemia injury. In cerebral ischemia–reperfusion injury (IRI), IRE1/TRAF2 axis activated MAPK and contributed to cell apoptosis both in vitro and in vivo [11]. Moreover, SAPK and P38 have also been reported to mediate myocardial and renal IRI [12, 13]. Liu et al*.* demonstrated that N-methyl-D-aspartate receptor-driven calcium inflow aggravates human cardiomyocyte apoptosis during the ischemia via P38 MAPK-mediated mechanism [14]. Moreover, it has been reported that USP49 overexpression could inhibit JNK1/2 activation and reduce human cardiomyocyte apoptosis, while JNK1/2 inhibitor treatment (SP600125) suppressed USP49 knockdown-induced JNK1/2 activation and cardiomyocyte apoptosis [15]. For renal IRI, renal tubular transient receptor potential ankyrin 1(TRPA1) is a potential oxidative stress sensor, which could regulate the activation of the MAPK/NF-κB signaling and production of IL-8, and aggravate renal tubular cell injury after HR [16].

Several clinical trials have also been conducted to investigate the role of MAPK inhibitors in either cardiac or renal IRI, but lack of studies in HIRI. D Talmor et al*.* first detected significant activation of ERK1/2, P38, and SAPK in human heart tissues during coronary artery bypass grafting [17]. It was also confirmed that P38 MAPK-stimulated inflammation contributes to atherogenesis, plaque instability, and maladaptive processes during myocardial infarction in another clinical trial [18]. A phase 2 trial indicated that, losmapimod, a P38 MAPK inhibitor, was indicated in phase 2 trials to reduce inflammation and alleviate cardiac IRI [19]. Compound α-ketoacid tablets have been reported to protect against IRI-induced renal injury and fibrosis by inhibiting NF-KB and MAPK signaling pathways [20].

As for HIRI, Amersi et al*.* reported carbon monoxide in preventing HIRI through inhibiting P38 MAPK ex vivo [21]. It has been found that astaxanthin, a carotenoid with strong antioxidant properties found in freshwater microalgae as well as marine organisms, inhibited apoptosis and autophagy in mice HIRI by reducing the ROS/MAPK pathway activity [22]. The role of MAPK signaling pathways in HIRI has been clarified, and targeting MAPK-related molecules is thought to be a potential therapeutic strategy for ameliorating HIRI, but still lacks of effective application of direct MAPK inhibitors to prevent or treat HIRI [10]. Meanwhile, regulators of the MAPK pathway have also been selected to be potential therapeutic targets for HIR treatment.

Cysteine-serine-rich nuclear protein 1 (CSRNP1), termed axin1 up-regulated 1 (AXUD1), is a nuclear protein with high conservation and plays critical roles in biological processes such as cell proliferation and viability [23]. In Drosophila, Glavic et al. first demonstrated that increased CSRNP1 expression interferes with cell cycle progression during mitosis through the disruption of Cdk1 activity, resulting in apoptosis in the presence of JNK [24]. Consistently, siRNA-mediated *CSRNP1* down-regulation resulted in reduced JNK phosphorylation and apoptosis in compressed human primary cementoblasts [25]. More recently, studies have identified that *CSRNP1* was one of the most relevant genes to organ injuries including hypoxic-ischemic encephalopathy and non-alcoholic fatty liver disease (NAFLD) via sequencing and bioinformatic strategies, but lack of further investigation [26, 27]. Notably, the underlying mechanism mediated by CSRNP1 in HIRI remains unclear.

The present study demonstrated that increased CSRNP1 expression was observed in the hepatocyte hypoxia-reoxygenation (HR) model, mouse HIRI model, and human liver samples after transplantation. Hepatocyte apoptosis was significantly suppressed by CSRNP1 inhibition, which is highly associated with MAPK signal inactivation both in vitro and in vivo. Thus, CSRNP1 could be a potential therapeutic target to ameliorate HIRI in an MAPK-dependent manner during LT.

## Results

### Identification of DEGs

**Fig. 1:**
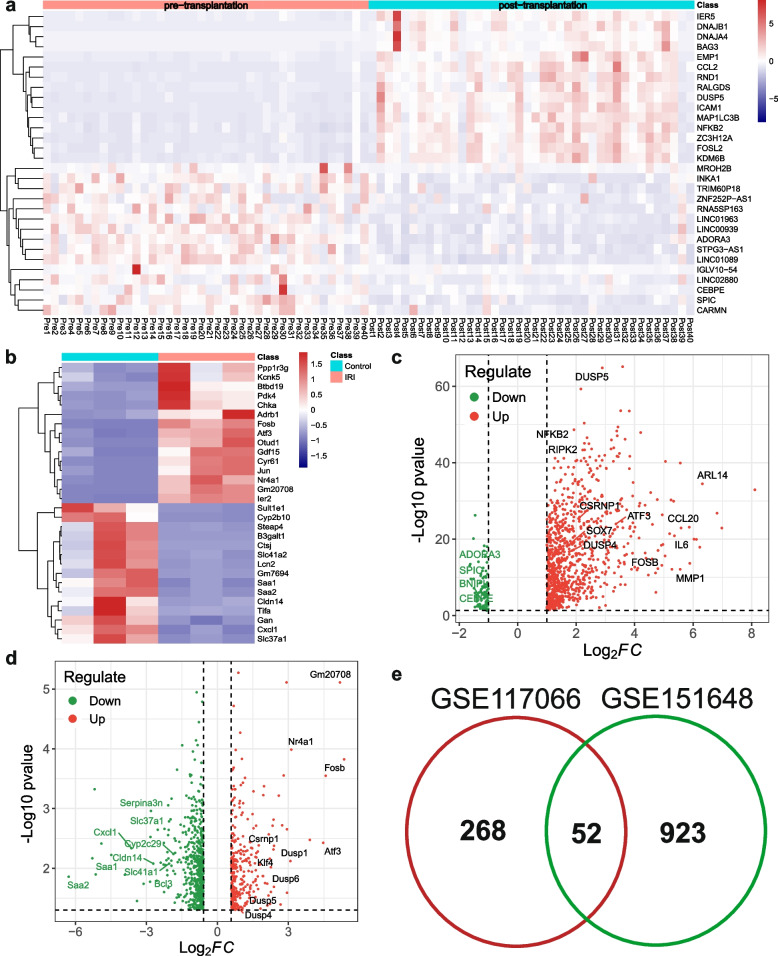
Identification of DEGs in the GSE151648 and GSE117066 cohort. **a** Heatmap displaying some of the DEGs between pre-LT and post-LT samples in the GSE151648 dataset. **b** Heatmap displaying some of the DEGs between HIRI samples and negative controls in the GSE117066 dataset. **c** Volcano plot displaying all the DEGs between pre-LT and post-LT samples in the GSE151648 dataset. **d** Volcano plot displaying all the DEGs between HIRI samples and negative controls in the GSE117066 dataset. **e** Venn plot showing the commonly upregulated genes in both the GSE151648 and GSE117066 cohort

The DEGs in the GSE151648 cohort were identified using the following criteria: |log FC|> 1 and *P* < 0.05. In total, 975 genes were upregulated while only 85 genes were downregulated in post-transplantation samples. The DEGs in the GSE117066 dataset were available in the GEO database (https://www.ncbi.nlm.nih.gov/). We selected the top 15 upregulated genes and the top 15 downregulated genes in each cohort, whose expression was used to draw heatmaps (Fig. 1a and b). Additionally, we used a Volcano plot to visualize the DEGs in the two datasets (Fig. 1c and d). Considering that the majority of DEGs in the GSE151648 cohort were upregulated in the post-transplantation group, we assumed that some of them might be involved in the pathophysiological process of LT and may become potential therapeutic targets to prevent or ameliorate post-transplant complications such as HIRI. We further selected the shared upregulated genes between the GSE151648 and GSE117066 cohorts and a total of 52 commonly-upregulated genes were identified (Fig. 1e). The DEGs existing in both the GSE151648 cohort and GSE117066 cohort might play a similar role in inducing or accelerating HIRI.

### Identification of enriched pathways

**Fig. 2:**
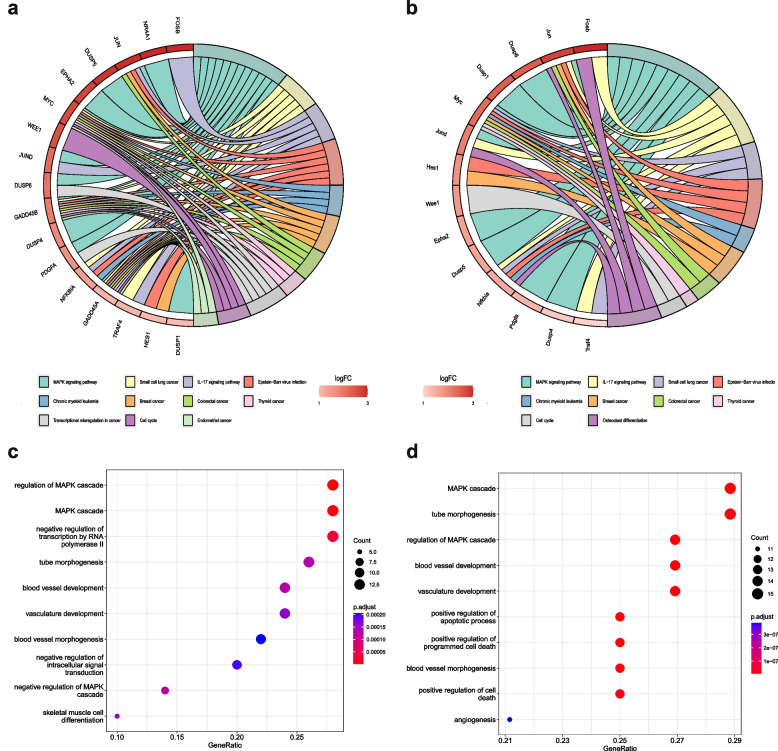
KEGG and GO analysis of the commonly upregulated genes in different species. **a** Chordal graph showing the enriched GO pathways in humans. **b** Chordal graph showing the enriched GO pathways in mice. **c** Bubble plot showing the enriched KEGG pathways in humans. **d** Bubble plot showing the enriched KEGG pathways in mice

We first performed GO analysis to enrich pathways among the 52 shared upregulated genes. The results suggested that these commonly upregulated genes were enriched in signaling pathways including MAPK and blood vessel formation (Fig. 2a and b). Additionally, KEGG analysis was also applied and we discovered that the MAPK pathway was also enriched, together with some cancer-related pathways including breast cancer and small cell lung cancer (Fig. 2c and d). The above results indicated that the MAPK pathway is of great importance in the regulation of HIRI during and after the process of LT.

### Identification of CSRNP1 as an MAPK-associated gene and a potential therapeutic target for HIRI

**Fig. 3:**
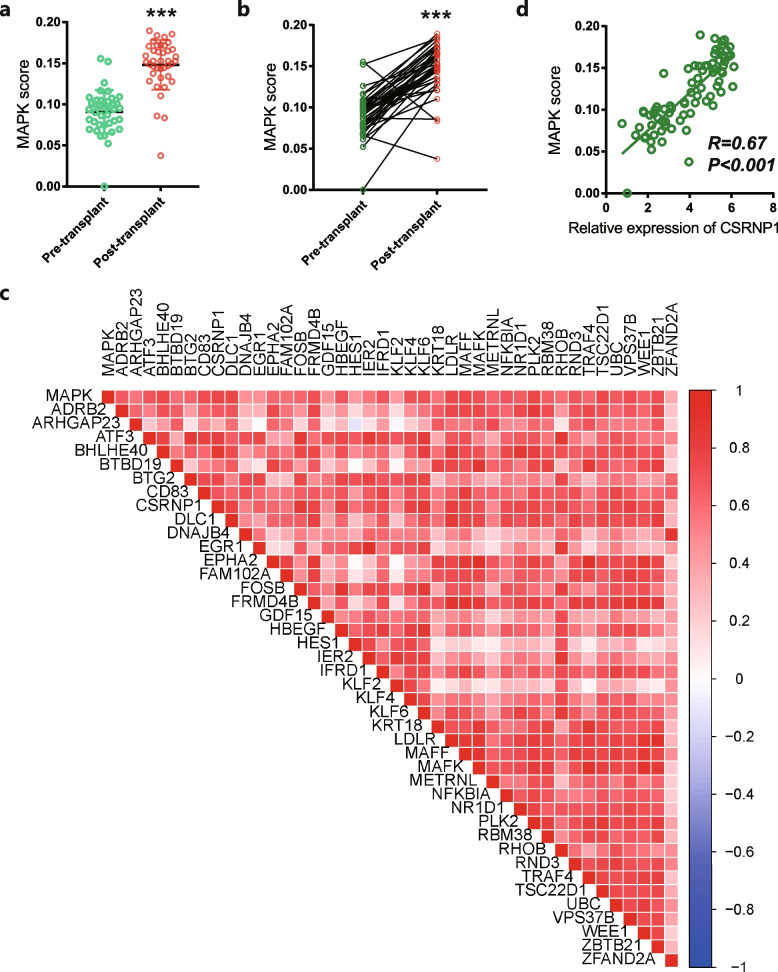
Identification of CSRNP1 as a potential therapeutic target in the datasets.** a** MAPK scores of pre-transplant and post-transplant samples in the GSE151648 dataset. **b** Comparison of the MAPK score before and after LT in the GSE151648 dataset. **c** Correlation heatmap displaying the Pearson correlation coefficient between the MAPK score and relevant genes. **d** Correlation plot displaying the Pearson correlation coefficient between the MAPK score and *CSRNP1* expression. ***P < 0.001 *vs* pre-transplant group. LT: liver transplantation

We calculated the MAPK pathway activity of the samples in the GSE151648 cohort. The results suggested that significant activation of the MAPK pathway was observed in post-transplantation samples (Fig. 3a). The paired t-test also consistently showed that higher MAPK activity scores were detected in the post-transplant group than pre-transplant (Fig. 3b). However, 41 of the 52 genes were not included in the gene list of the MAPK pathway. We next performed Pearson analysis to identify the correlation between the expression of these genes and MAPK scores. Importantly, we found that the majority of these genes demonstrated positive correlations with MAPK scores (Fig. 3c). Among these genes, *CSRNP1* had the highest correlation coefficient with the MAPK score (Fig. 3d).

### CSRNP1 is significantly upregulated in vitro and in vivo

**Fig. 4:**
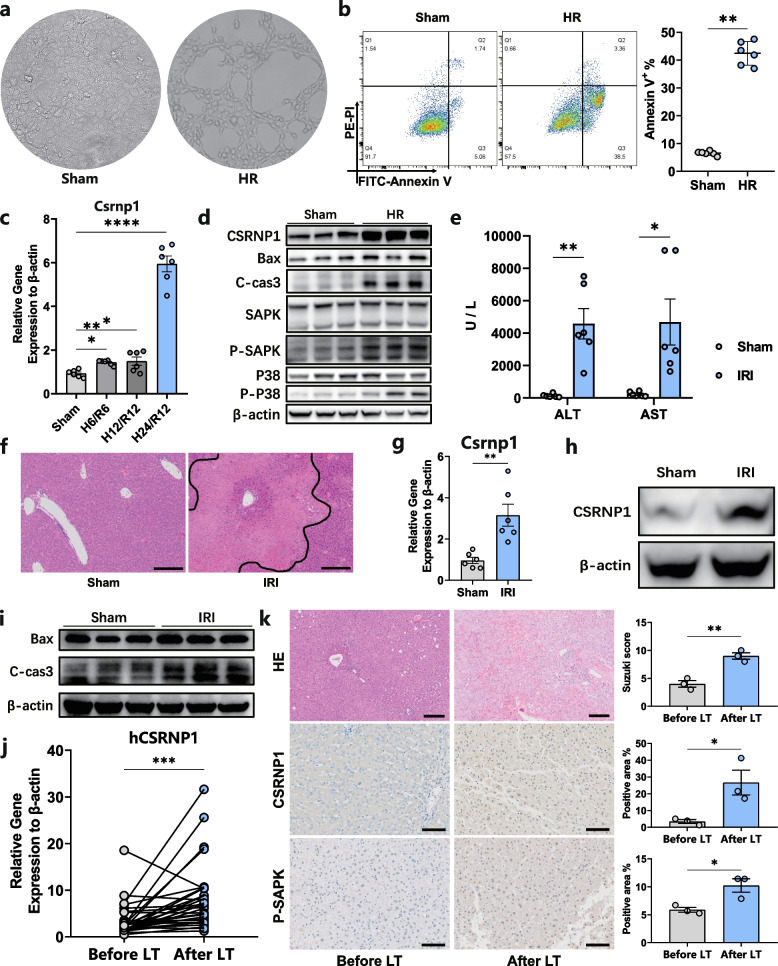
Identification of CSRNP1 upregulation in vitro and in vivo. **a** Representative photographs of the cell HR model. **b** Apoptosis of AML-12 cells was assessed by flow cytometry analysis *n* = 6). **c** Relative mRNA levels of *CSRNP1* assessed by qRT-PCR in AML-12 cells of the sham and different HR groups (H: hypoxia; R: reoxygenation) (*n *= 6). **d** Western blot showing the levels of Bax, cleaved caspase 3, SAPK, phosphorylated SAPK, p38, phosphorylated p38, and CSRNP1 in AML-12 cells of the sham and HR group. **e** Serum ALT and AST levels in mice after sham surgery or HIRI (*n* = 6). **f** Representative H&E staining images and necrotic areas of mouse liver lobes after HIRI (scale bar = 200 μm). **g** Relative mRNA levels of *CSRNP1* were assessed by qRT-PCR in liver tissues from sham and HIRI mice (*n* = 6). **h** Western blot showing the levels of CSRNP1 in liver tissues of sham and HIRI groups. **i** Western blot showing the levels of Bax and cleaved caspase 3 in liver tissues of sham and HIRI groups. **j** Relative mRNA levels of *CSRNP1* were assessed by qRT-PCR in liver tissues before and after LT (*n* = 32). **k** Representative images of IHC for CSRNP1 protein in human liver samples before and after LT (scale bar = 100 μm).** l** Representative H&E staining images of human liver samples before and after LT (scale bar = 100 μm) (*n* = 3). *P < 0.05, **P < 0.01, ***P < 0.001, ****P < 0.0001. IRI: ischemia–reperfusion injury; HR: hypoxia-reoxygenation; LT: liver transplantation

In order to further confirm the analytic data, we first constructed an in vitro cell HR model to imitate the process of HIRI in vivo. The AML-12 cell line was incubated under hypoxic conditions for 24 h and then changed to a normal cell incubator with oxygenation for another 12 h. As shown in Fig. 4a, a great proportion of the cells died after HR, which showed a sparse distribution of live cells. Flow cytometry of cell apoptosis revealed a significantly higher ratio of Annexin^+^ cells in the HR group (Fig. 4b). Gene expression of *Csrnp1* in cells under HR was markedly higher (Fig. 4c), and the protein level of CSRNP1 was also upregulated (Fig. 4d). Consistent with flow cytometry, higher expression of Bax and cleaved caspase 3 were also detected in cells after HR, which indicated severe cell apoptosis (Fig. 4d). Moreover, markedly higher protein expression of phosphorylated SAPK and P38 were also detected in the HR group, which demonstrated overactivation of the MAPK signal pathway related to cell stress and apoptosis (Fig. 4d). The above results indicated that in cell HR models, CSRNP1 is significantly upregulated accompanied with activation of MAPK pathway.

To verify the expression of CSRNP1 in mouse HIRI models, the vessels in the left 70% lobes were clamped for 90 min and then the clamp was removed for 6 h of blood reperfusion. In the HIRI group, a marked increase of ALT and AST levels was observed compared to the sham group (Fig. 4e). As a further assessment of liver injury, histopathology revealed both inflammatory cell infiltration and severe necrosis in the HIRI group (Fig. 4f). It was also found that Csrnp1 mRNA expression was significantly increased in the HIRI group (Fig. 4g). As expected, expression of CSRNP1 was also consistently increased following HIRI (Fig. 4h). The expression of apoptosis-related proteins, Bax and cleaved caspase 3, was also significantly increased following HIRI (Fig. 4i). To further confirm the in vitro and in vivo results, we examined the gene expression of *CSRNP1* in human liver samples collected from recipients before and after LT from our center. Consistently, CSRNP1 expressed at a significantly higher level after LT both at gene and protein levels while the histological necrosis was also obvious (Fig. 4j-k). The above results indicated that CSRNP1 may exert an important role in regulating HIRI.

### Inhibition of CSRNP1 exerted a protective effect on hepatocyte HR model via MAPK signaling pathway

**Fig. 5:**
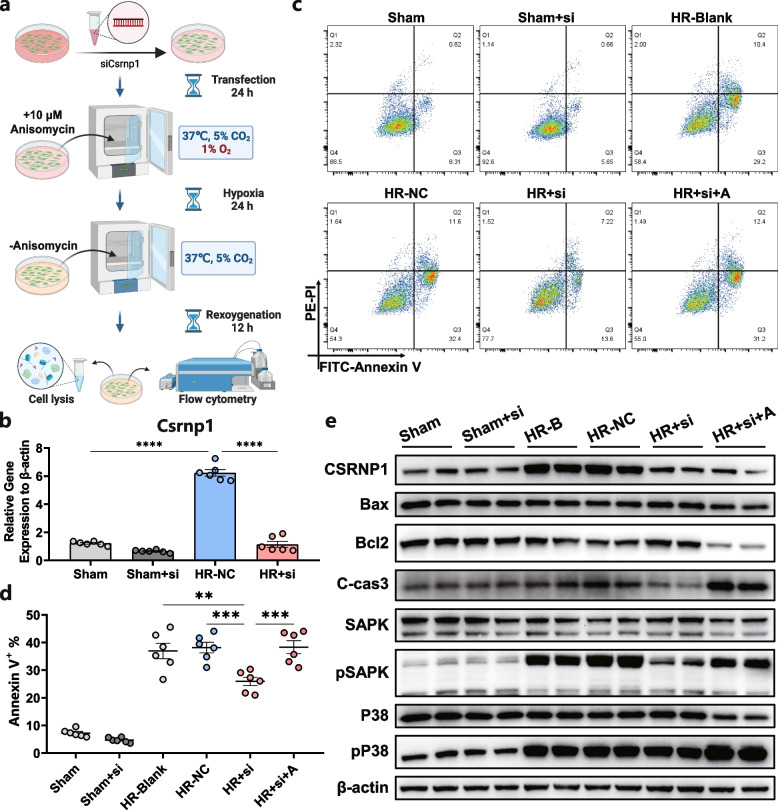
CSRNP1 knockdown exerted a protective effect on hepatocytes after HR via MAPK signaling pathway in vitro. **a** Schematic of the workflow (Created with BioRender.com, Agreement number: DU26KZDY58). **b** Relative mRNA levels of *CSRNP1* were assessed by qRT-PCR in AML-12 cells of the sham and HR group with or without transfection of *CSRNP1*-siRNA (*n* = 6). **c** Apoptosis of AML-12 cells after HR was assessed by flow cytometry (*n* = 6). **d** Quantification charts of flow cytometry in Fig. 5c (*n* = 6). **e** Representative blots showing the protein level of CSRNP1; apoptosis related Bax, Bcl2 and cleaved caspase3; MAPK related SAPK, phosphorylated SAPK, P38 MAPK, and phosphorylated P38 MAPK; and internal control protein β-actin. **P < 0.01, ***P < 0.001, ****P < 0.0001. HR: hypoxia-reoxygenation; si: CSRNP1-siRNA; B: Blank; NC: negative control; A: Anisomycin (JNK activator)

In order to clarify the role of CSRNP1 in hepatocyte HR, siRNA was designed to knock down *Csrnp1* gene expression in hepatocyte HR model (Fig. 5a-b). Flow cytometry revealed a lower ratio of cell apoptosis in the *Csrnp1* knockdown group after HR (Fig. 5c-d). As shown in Fig. 5e, suppressing Csrnp1 also leads to decreased expression of Bax and cleaved caspase 3, but increased levels of antiapoptotic protein Bcl2. Moreover, activated MAPK-related proteins phosphorylated SAPK and phosphorylated P38 MAPK were both reduced after *Csrnp1* knockdown (Fig. 5e). It has been reported that suppressing MAPK activation (either P38 or SAPK) could effectively reduce apoptosis and ameliorate HIRI [10, 28, 29]. To further understand the direct link and causal relationship between CSRNP1 and MAPK, MAPK activator, Anisomycin, was added to the *Csrnp1* knockdown group before HR. As expected, the expression of MAPK-related proteins was upregulated and *Csrnp1* silencing-induced anti-apoptosis effect was eliminated (Fig. 5c-e). Therefore, these findings support the hypothesis that CSRNP1 accelerates apoptosis in HR-treated hepatocytes, and inhibition of CSRNP1 exerted a protective effect on the hepatocyte HR model via the MAPK signaling pathway.

### CSRNP1 knockdown ameliorates mouse HIRI

**Fig. 6:**
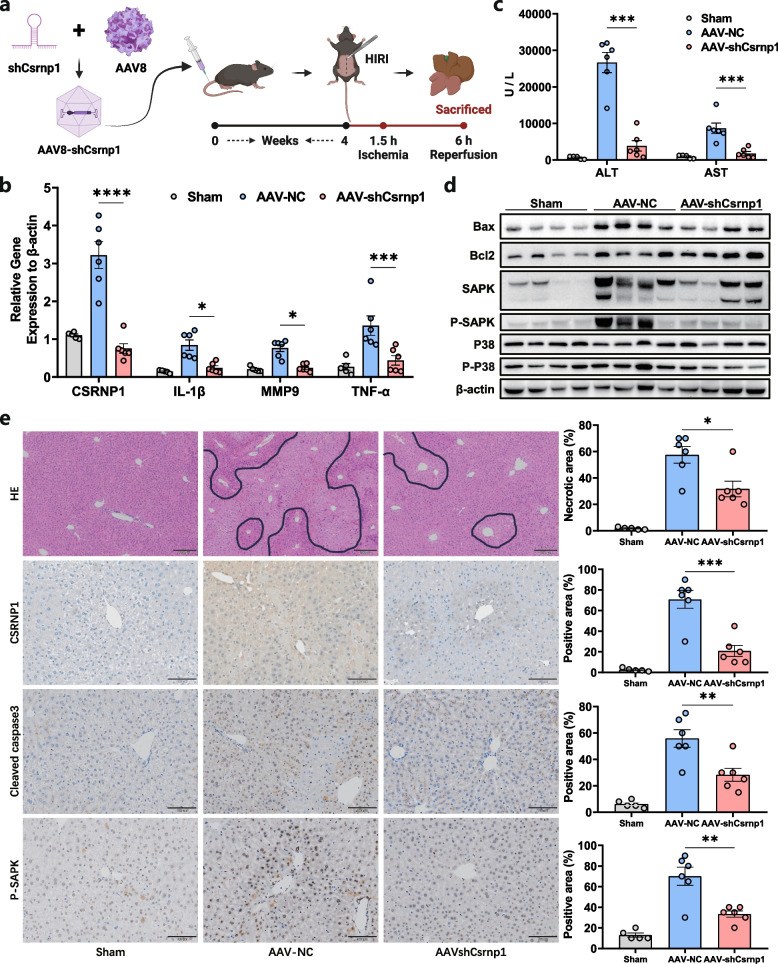
CSRNP1 Knockdown Ameliorates Mouse HIRI via MAPK Pathway. **a** Schematic of the workflow (Created with BioRender.com, Agreement number: VB26NQ5MCI). **b** Relative mRNA levels assessed by qRT-PCR in liver tissue of the sham (*n* = 5), AAV-NC (*n* = 6) and AAV-shCsrnp1 (*n* = 6) group. **c** Serum ALT and AST levels in mice after sham surgery or HIRI. **d** Western blot showing the levels of marker proteins related to apoptosis, MAPK signal pathway, and corresponding phosphorylated forms.** e** Representative H&E staining images (scale bar = 200 μm), IHC for CSRNP1, cleaved caspase3 and phosphorylated SAPK (scale bar = 100 μm). *P < 0.05, **P < 0.01, ***P < 0.001

Next, we investigate whether the inhibition of CSRNP1 could also affect HIRI in vivo. By intravenous injection of AAV8-sh*CSRNP1*, mouse liver expressed a significantly lower level of *CSRNP1* after four weeks (Fig. 6a and b). Liver injury was remarkedly reduced in *CSRNP1*-knockdown mice after HIRI (Fig. 6c). Relative mRNA expression of inflammatory and tissue injury markers including *Il-1β*, *Tnf-α,* and *Mmp9* were also lower in mice after knocking down *CSRNP1* in liver (Fig. 6b). Consistently, protein levels of apoptosis-related Bax and cleaved caspase3 were also down-regulated in mice with lower CSRNP1 expression after HIRI (Fig. 6d and e). Histology analysis revealed less necrosis and apoptosis in liver tissues after knocking down CSRNP1 (Fig. 6e). Mechanically, genetic interference of *CSRNP1* significantly inhibited the phosphorylation of the MAPK pathway especially P38 and SAPK (Fig. 6d and e). Together, these results indicated that CSRNP1 knockdown ameliorates mouse HIRI via inhibition of MAPK pathway activation.

### Delivering siNP attenuates mouse HIRI in vivo

**Fig. 7:**
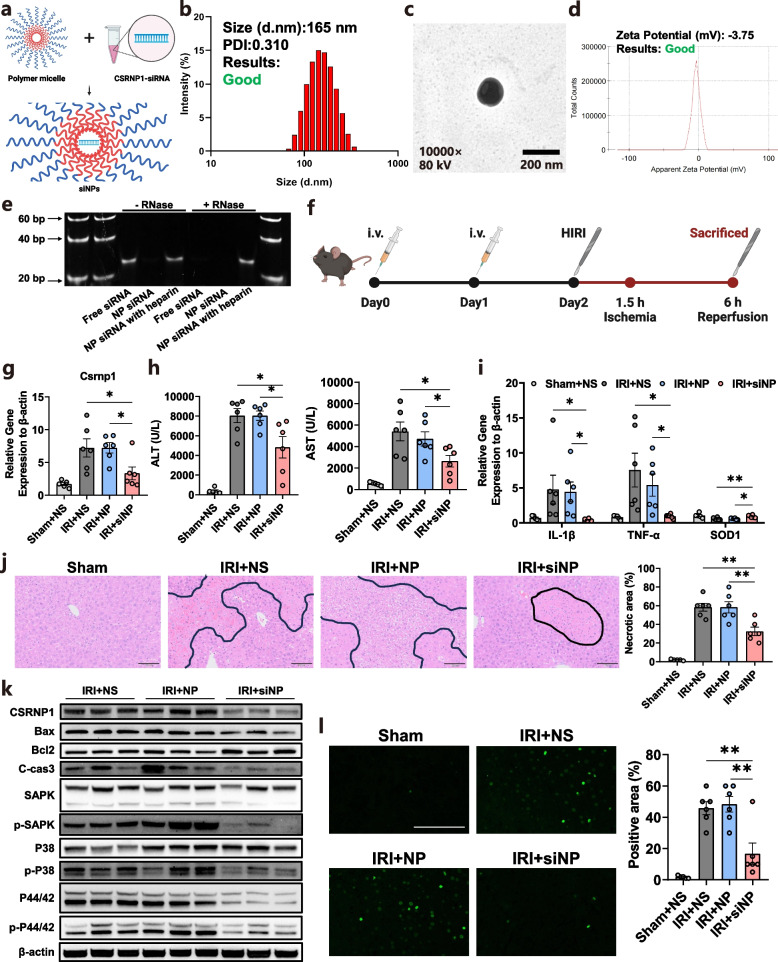
Delivering siNP attenuates mouse HIRI in vivo. **a** Schematic of workflow for construction of siNP (Created with BioRender.com, Agreement number: EX26KZEGAI). **b** The optimized size distribution of siNP is determined by dynamic light scattering (DLS). **c** Representative zeta potential of siNP determined by DLS. **d** Representative image of siNP under the transmission electron microscope (TEM). **e** RNase protection assay. **f** Schematic of workflow for treatment of siNP in mouse HIRI model (Created with BioRender.com, Agreement number: DB26NP7AXT). **g** Relative mRNA levels of *CSRNP1* were assessed by qRT-PCR in liver tissue of the Sham (*n* = 5), IRI-NS (*n* = 6), IIR-NP (*n* = 6), and IRI-siNP (*n* = 6) groups. **h** Serum ALT and AST levels in mice after sham surgery or HIRI. **i** Relative mRNA levels of *Il-1β*, *Tnf-α*, and *Sod1* assessed by qRT-PCR in mouse liver. **j** Representative H&E staining images (scale bar = 100 μm) and corresponding Suzuki score quantification. **k** Western blot showing the levels of marker proteins related to apoptosis, and MAPK signaling pathway. **l** Representative images of TUNEL staining among liver tissues from different groups (scale bar = 100 μm). *P < 0.05, **P < 0.01, ***P < 0.001

To further substantiate the inhibitory effect of CSRNP1 on HIRI and to mitigate the adverse effects associated with the viral vectors used in gene therapy, we employed DSPE-PEG-NHS nanoparticles (NP) to encapsulate *CSRNP1*-siRNA, thereby creating siRNA nanoparticles (siNP), as depicted in Fig. 7a. The siNP exhibited an average size peaking at approximately 160 nm (Fig. 7b and c). To avert the aggregation of siNPs with proteins in the bloodstream, negative zeta potential is advantageous. As demonstrated in Fig. 7d, the zeta potential of the siNPs approached zero, indicating their suitability for in vivo use. An RNase protection assay was conducted to verify the encapsulation of the siRNA, and heparin was utilized to release the siRNA from siNP. The siRNA was shielded from RNase-induced degradation by the NP and was successfully released following the addition of heparin, as shown in Fig. 7e.

Subsequently, we administered normal saline (NS), nanoparticles (NP), or siRNA nanoparticles (siNP) to mice for two consecutive days prior to either HIRI or sham surgery, as illustrated in Fig. 7f. Gene expression analysis revealed a significant reduction of *CSRNP1* level in mouse liver samples from the IRI + siNP group, with no detectable differences between the NS and NP groups (Fig. 7g). Liver function exhibited consistent alterations with *CSRNP1* gene expressions across the groups, with significant deterioration observed in both the NS and NP groups, but apparent amelioration in mice treated with siNP (Fig. 7h).

The intra-hepatic levels of pro-inflammatory genes, including *Il-1β* and *Tnf-α*, were markedly declined in mice injected with siNP, while the expression of antioxidant gene *Sod1* was upregulated to levels comparable to the Sham group (Fig. 7i). Histological evaluations of liver damage, according to the necrotic, also indicated a diminution in the siNP group (Fig. 7j). A notable reduction in intrahepatic apoptosis was observed, concomitant with a significant decrease in CSRNP1 expression levels, as shown in Fig. 7k. The TUNEL staining of liver tissues also indicated a significantly lower level of cell apoptosis after administration of siNP (Fig. 7l). Notably, the MAPK signaling pathway was markedly suppressed following the siNP-induced genetic inhibition of *CSRNP1*, which elucidated the underlying mechanism by which CSRNP1 contributes to HIRI (Fig. 7k). Collectively, these findings demonstrate that the in vivo delivery of siNP to inhibit *CSRNP1* is both safe and efficacious, presenting a potential therapeutic target for mitigating graft injury during LT in the future.

## Discussion

HIRI occurs in several clinical processes, including LT, trauma, major hepatectomy, and hemorrhagic shock [30]. Although various surgical techniques have improved post-transplant outcomes on a large scale, postoperative liver dysfunction and failure are still caused primarily by HIRI, especially after LT [31]. Considerable effort has been made to understand the mechanisms responsible for HIRI. However, it is still poorly understood how ischemia and reperfusion regulate liver function at the molecular and cellular levels [4].

Bioinformatics research has been widely used in the screening of signaling pathways and key molecular targets. In cancer research, such as in breast cancer and non-small cell lung cancer, researchers are focusing on analyzing large amounts of biological data at scale, which could help find valuable prognostic, predictive, and even therapeutic targets [32, 33]. Nevertheless, the utilization of bioinformatics research has also been applied in the field of non-cancer studies including HIRI, which could reveal the underlying mechanisms of organ repair and regeneration [34]. The transcriptional profiles of single cells within liver transplanted organs have recently been systematically and comprehensively established, integrated with bioinformatics research, it is possible to find novel resolution for ameliorating HIRI in the future [35].

In this study, we identified that the most significantly upregulated DEGs in both human LT samples and mouse HIRI model were enriched in the MAPK signal pathway, indicating the importance of MAPK in modulating HIRI. Multiple members of the MAPK family have been shown in previous studies to inhibit apoptosis, thus reducing IRI in organs. Most cell types are simultaneously activated by JNK/SAPKs and P38 MAPKs when exposed to external stresses, but their exact roles in inducing apoptosis are still unclear [36].

In cardiac IRI, JNK activation acts as a pro-apoptotic component by regulating BCL2-associated cell death phosphorylation, which reverses heart injury by inhibiting JNK activation [37]. In kidney, JNK and p38 were both activated during IRI, and trans-cinnamaldehyde was utilized to inhibit JNK/P38 MAPK and alleviate renal injury [12]. Recently, integrated omics revealed MAPK signaling as a downstream for inducing HIRI, and could be modulated by toll-interacting protein expression in mice [38]. Therefore, the MAPK signaling pathway is consistently activated in different organ injuries, differences may occur in the phosphorylation of specific marker genes.

Next, we demonstrated that CSRNP1 was the most significantly correlated with MAPK signal pathway by bioinformatical analysis, then confirmed its upregulation among the hepatocyte HR model, mouse HIRI model, and human liver samples after transplantation. Previous studies did not reveal any direct correlation between CSRNP1 and HIRI. CSRNP1 has only been identified as a key gene associated with neonatal hypoxic-ischemia brain damage by RNA sequencing and single-nucleotide polymorphism screening, and also a candidate target for hypoxic-ischemic encephalopathy therapy, but lacks further investigations [26]. In our study, a significant protective effect on HIRI was detected after inducing CSRNP1 inhibition by either AAV or nanoparticle delivery in vivo, indicating CSRNP1 as a potential therapeutic target to reduce organ injury during LT.

Furthermore, our study identified that inhibition of CSRNP1 is able to down-regulate MAPK signal activation in the mouse HIRI model. Mechanically, CSRNP1-dependent cell apoptosis was mainly linked to the induction and activation of JNK/SAPK and P38 that respond to various exogenous stimuli [39]. In Drosophila, overexpression of CSRNP1 induced SAPK-dependent apoptosis [24]. Consistently, CSRNP1 was also reported to induce human cementoblast apoptosis by upregulating the phosphorylation of SAPKs [25]. The phosphorylation of SAPK and P38 leads to translocation to the nucleus where they are phosphorylated and transactivate several transcription factors leading to increased expression of pro-apoptotic genes [40]. However, the concise correlations between CSRNP1 upregulation and MAPK activation as well as the specific underlying mechanisms of CSRNP1 inhibition down-regulating MAPK signaling pathway have not been clarified in this study, and require further investigation. In addition, as a nuclear protein involved in critical biological processes, the comprehensive safety and off-target effect evaluations of CSRNP1-related therapeutic strategies are paramount to ensure viability and feasibility. So whether CSRNP1 could be a potential therapeutic target for ameliorating HIRI required further investigations in big animal studies and clinical trials.

**Fig. 8:**
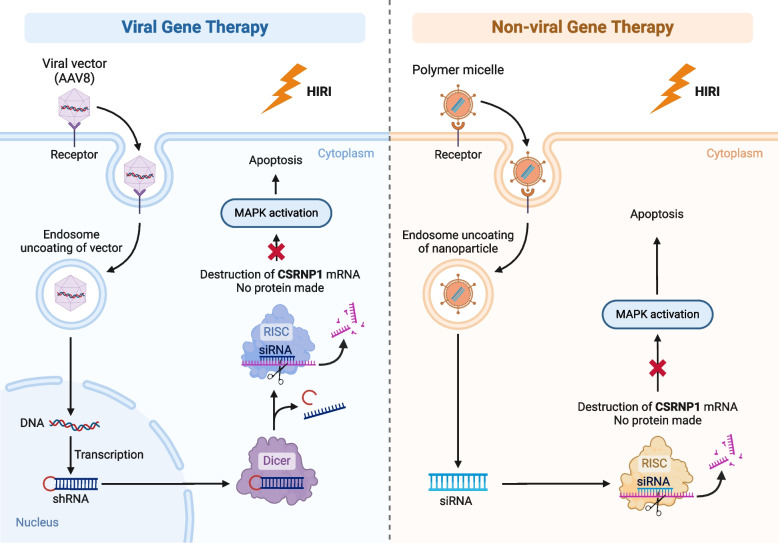
Targeting CSRNP1 to ameliorate ischemia–reperfusion injury during liver transplantation. This graph illustrates the potential therapeutic strategies targeting CSRNP1, including viral and nanomedical delivery of genetic interfering tools, to potentially alleviate graft injury via inhibiting MAPK-dependant hepatocyte apoptosis. (Created with BioRender.com, Agreement number: IT26KZELCN)

In conclusion, the present study demonstrated that CSRNP1 was upregulated in the cell HR model, mouse HIRI model, and human LT samples. Hepatocyte apoptosis was significantly suppressed by CSRNP1 inhibition in an MAPK-dependent manner via genetic knockdown in vitro*, and* both viral and non-viral (nanoparticles) gene therapies i*n vivo* (Fig. 8). In this regard, CSRNP1 could function as a potential therapeutic target to improve HIRI.

## Materials and methods

### Data acquisition and processing

The expression matrix used in this study was obtained from the GEO database (https://www.ncbi.nlm.nih.gov/). The GSE151648 dataset contains the RNA-sequencing data of 40 patients who underwent orthotopic LT. The differentially expressed genes (DEGs) between pre-transplantation liver samples and post-transplantation liver samples were identified using the “edgeR” package. The fragments per kilobase of exon model per million mapped fragments value of 3 liver samples with IRI and relevant negative controls were obtained from the GSE117066 dataset for further analysis.

### Functional enrichment analysis

For Gene Ontology (GO) analysis and Kyoto Encyclopedia of Genes and Genomes (KEGG) analysis, we used the DEGs existing in both the GSE151648 and GSE117066 datasets to identify relevant enriched pathways. Genes symbols were converted into ENTREZ ID.

### ssGSEA

Single-sample gene set enrichment (ssGSEA) can evaluate the activities of signaling pathways at a single-sample level. We downloaded the gene list from the msigDB database (http://www.gsea-msigdb.org/gsea/msigdb/collections.jsp#H). The ssGSEA scores of each group were calculated using the “GSVA” package.

### PPI Network

To visualize the interactions among proteins, the STRING database (https://cn.string-db.org/) was applied to construct a PPI network. An interaction can be constructed if the interaction score between two proteins is more than 0.4.

### Animals and murine models of HIRI

All experiments were conducted according to the Ethics Committee of Zhejiang University. The animal protocol was designed to minimize pain or discomfort to the animals.

Six to eight-week-old C57BL/6 mice were purchased from Hangzhou Medical College (Hangzhou, China). Prior to experimentation, animals were acclimatized in isolated ventilated cages under specific pathogen-free conditions (23 °C, 12 h/12 h light/dark, 50% humidity, free access to food and water) for 1 week. Mice were around 8 weeks old at the time of surgery. HIRI was performed as described previously [41]. In summary, the arterial and portal vessels of the left and median lobes were occluded for 90 min, followed by the removal of the clamp to allow blood reperfusion. The animals were then situated on a heating pad regulated at 37 °C until they fully regained consciousness. Six hours post-reperfusion, all animals were humanely euthanized via an overdose of barbiturates (intravenous injection of 150 mg/kg pentobarbital sodium) to facilitate the collection of blood and tissue samples.

### Adeno-associated virus construction and infection

Adeno-associated virus serotype 8 (AAV8) was employed to engineer short hairpin RNAs (shRNAs) directed against the CSRNP1 gene, sourced from GeneChem in Shanghai, China. The recombinant virus, at a concentration of 1.5 × 10^11^ viral genomes, was diluted in 0.2 mL of saline solution and administered via the tail vein to the mice. The control group received an identical volume of saline solution infused with a negative control virus. Four weeks following the injection, the mice underwent HIRI procedures.

### Liver function

Serum concentrations of alanine aminotransferase (ALT) and aspartate aminotransferase (AST) were measured utilizing an automatic biochemical analyzer (Chemray 800, Rayto, Shenzhen, China), in strict accordance with the guidelines provided by the manufacturer.

### Histology and Immunohistochemistry (IHC)

Paraffin-embedded liver specimens, sectioned at 5 µm, underwent deparaffinization with xylene, followed by rehydration through a graded ethanol series, and were subsequently stained with hematoxylin and eosin (H&E). Photographic documentation was achieved using a light microscope (Olympus, Tokyo, Japan).

For the immunohistochemical analysis, the sections were placed in a repair box filled with citric acid antigen retrieval buffer (pH 6.0) for antigen retrieval, followed by treatment with 3% hydrogen peroxide to quench endogenous peroxidase activity. Subsequently, they were blocked with 10% bovine serum albumin (BSA) for 1 h at 37 °C. The sections were then incubated with anti-CSRNP1 primary antibodies (ABclonal, #A7130) overnight at 4 °C, and subsequently with appropriate biotinylated secondary antibodies (ServiceBio, #G1213, #G1214) for 1 h at room temperature. Subsequently, the sections were incubated with a newly prepared DAB color-developing solution (ServiceBio, #G1212) and visualized under a microscope.

### Cell culture

Alpha mouse liver 12 (AML-12) cells (normal mouse hepatocytes) were cultured at 37 °C in a humidified atmosphere with 5% CO_2_ in DMEM: F12 medium (Sigma, #D8437) supplemented with 10% heat-inactivated fetal bovine serum (Multicell, #086–150), 1% penicillin–streptomycin (Biosharp, #BL505A), 1% insulin-transferrin-selenium (ITS, Procell, #PB180430) and 40 ng/mL dexamethasone (MCE, #PB180430).

In order to imitate the process of HIRI in vitro, AML-12 cells were changed to non-serum culture media and incubated under hypoxic conditions (37 °C, 5% CO_2,_ and 1% O_2_) for 6, 12, or 24 h, respectively, and then changed to a normal cell incubator with oxygenation for another 6 or 12 h. In order to evaluate the effect of MAPK activation on Csrnp1 silencing-induced anti-apoptosis, Anisomycin (JNK activator, 10 μM) was added to the medium before HR.

### Transfection

To inhibit CSRNP1, siRNA targeting *CSRNP1* (sense: 5’-CGAGUGGAAUUCAAU-CAGA-3’; antisense: 5’-UCUGAUUGAAUUCCACUCG-3’) were designed and synthesized by Tsingke Biotech (Beijing, China). *CSRNP1* siRNA or scramble siRNA was transfected into cells with jetPRIME transfection reagent (Polyplus, #101,000,046) for 24 h and then the cells were HR-treated.

### Quantitative RT–PCR

Total RNA was extracted using the MolPure RNA Kit (Yeasen, China) according to the instructions. RNA reverse transcription was performed on an S1000 Thermal Cycler (Bio-Rad, USA) using Hifair cDNA Synthesis SuperMix (Yeasen, China). Real-time quantitative PCR (RT-qPCR) was performed on Light Cycler 480 (Roche) with Hieff SYBR Green Master Mix (Yeasen, China). Beta-actin was utilized as an endogenous control. All primers used for RT-qPCR were synthesized by Tsingke Biotech (Beijing, China). The sequences are listed in Table 1.

**Table 1:** Primer sequences of RT-qPCR

Targets	Forward	Reverse
*mβ-Actin*	GTGACGTTGACATCCGTAAAGA	GCCGGACTCATCGTACTCC
*mGapdh*	AGGTCGGTGTGAACGGATTTG	GGGGTCGTTGATGGCAACA
*mSod1*	AACCAGTTGTGTTGTCAGGAC	CCACCATGTTTCTTAGAGTGAGG
*mSod2*	AGACCTGCCTTACGACTATGG	CTCGGTGGCGTTGAGATTGTT
*mCsrnp1*	TCCAGAGTTTCACTCCCCC	GGCACCGTGGGAAATAGTAGA
*mIl-1β*	GAAGAGCCCATCCTCTGTGA	GGGTGTGCCGTCTTTCATTA
*mMmp9*	GGACCCGAAGCGGACATTG	CGTCGTCGAAATGGGCATCT
*mTnf-α*	CCTGTAGCCCACGTCGTAG	GGGAGTAGACAAGGTACAACCC
*hβ-ACTIN*	GGCACCCAGCACAATGAAG	CCGATCCACACGGAGTACTTG
*hCSRNP1*	CCTGCCTGACCGTGACTT	AGCCCGCTTCAGGATAGAC

### Western blot

Total proteins were extracted using a cell lysis buffer (Fdbio, China). The protein concentration was determined by a BCA protein assay (Beyotime, China). Samples containing equal amounts of proteins were separated by 4%-20% sodium dodecyl sulfate polyacrylamide gel electrophoresis and were transferred onto a PVDF membrane (Millipore, USA), which was later blocked with 5% BSA and incubated with specific primary antibodies at 4 °C overnight. The membranes were then washed with TBST and incubated with an HRP-conjugated secondary anti-rabbit or anti-mouse antibody for 1 h at room temperature and visualized using ECL detection kits (Fdbio, China). Primary antibodies included Bax (1:1000, Cell Signaling Technology, #2772), Bcl2 (1:1000, Abcam, #ab182858), Cleaved caspase 3 (1:1000, Cell Signaling Technology, #9664), p44/42 MAPK (Erk1/2) (1:1000, Cell Signaling Technology, #4695), SAPK/JNK (1:1000, Cell Signaling Technology, #9252), p38 MAPK (1:1000, Cell Signaling Technology, #8690), Phospho-p44/42 MAPK (1:2000, Cell Signaling Technology, #4370), Phospho-SAPK/JNK (1:1000, Cell Signaling Technology, #4668), Phospho-p38 MAPK (1:1000, Cell Signaling Technology, #4511), and β-Actin (1:1500, ABclonal, #AC006).

### Apoptosis evaluation

Annexin V-FITC/PI detection kit (Multi Sciences, #AT101C) was used to perform flow cytometry analysis to assess the apoptosis of AML-12 cells according to the kit instructions and a previous report [42]. Briefly, the cells cultured in 6-well plates were trypsinized, rinsed with PBS, and re-suspended in 300 μL of binding buffer supplemented with 3 μL annexin V-FITC and 6 μL PI in darkness for 5 min on ice. Immediately after staining, the samples were analyzed by flow cytometry (BD, USA). The annexin V − /PI − cells were identified as viable; annexin V + /PI − as early-apoptotic; annexin V + /PI + as late-apoptotic; and annexin V − /PI + as cell debris.

### Preparation of nanoparticles containing siRNA

An aqueous siRNA solution was dissolved in 25µL of RNase-free water through sonication for 60 s within an ice bath. Subsequently, 1,2-distearoyl-sn-glycero-3-phosphoethanolamine-N-[hydroxyl succinimidyl (polyethylene glycol-5000)] (DSPE-PEG-NHS) was homogenized in 0.5 mL of chloroform. The two solutions were then fused in chloroform via sonication (90W for 1 min), while meticulously maintaining the temperature at 0 °C within an ice bath. This blend was gradually introduced to 3 mL of the solvent using a rotary evaporator set at 400 revolutions per minute. The concoction was then transferred to a 50 mL round-bottom flask and concentrated under reduced pressure using a rotary evaporator until the volume was reduced to 3 mL. The resulting solution underwent dialysis for three days (molecular weight cutoff = 10,000 Da) and was subsequently lyophilized to yield the final product. To ascertain the average sizes and zeta potential, dynamic light scattering (DLS) was employed to evaluate the unencapsulated nanomicelles and the siRNA-containing nanoparticles (siNP). Concurrently, a transmission electron microscope (TEM) was utilized to corroborate the findings from the DLS analysis.

### RNase protection assay

To ascertain the successful encapsulation of the siRNA, we executed an RNase protection assay. Twenty pmol of free siRNA or siNP was incubated with an equal volume of 50 μg/mL RNase A for 4 h at 37 °C. Following the enzymatic treatment, identical quantities of siNPs were treated with a 5 mg/mL heparin sodium solution for ten minutes to displace the siRNA within the nanoparticles. Free siRNA, siNP, and heparin-treated siNP were subjected to electrophoresis on a 10% polyacrylamide gel (PAGE) at a steady voltage of 160 V for 30 min in 1 × TBE buffer. The gel was then stained by incubating in a 3 × GelRed aqueous solution for one hour under room temperature. Subsequently, the gel was rinsed twice with pure water and visualized under ultraviolet light.

### Collection of human samples

All samples of transplanted livers were obtained from Shulan Hospital (Hangzhou, China) with Institutional Review Board approval and in accordance with the Helsinki Declaration. Written informed consent was obtained from the patients. A total of 32 pairs of liver tissue samples were collected during the procurement (before LT) and after reperfusion (after LT).

### Statistical analysis

The majority of the statistical analyses in this study were performed by R software. DGEs were analyzed using the “edgeR” package. All results are shown as the mean ± SEM. GraphPad Prism version 8.0 software (GraphPad, CA, USA) was used for generating graphs and performing statistical analysis. Comparisons between the two groups were analyzed by the Student’s *t* test. One-way or two-way analysis of variance (ANOVA) was used for comparisons between multiple groups. A *p* value < 0.05 was considered statistically significant.

## Data Availability

The data presented in this study are available in the article. The datasets used or analyzed during the current study are available from the corresponding author upon reasonable request.
